# TikTok fitspiration and fitness ideal internalisation: gender differences in self-esteem and body satisfaction

**DOI:** 10.3389/fpsyg.2025.1578510

**Published:** 2025-09-17

**Authors:** Maria Limniou, Holly Duckett, Eleanor Mitchell

**Affiliations:** Department of Psychology, University of Liverpool, Liverpool, United Kingdom

**Keywords:** TikTok fitspiration, fit internalisation, physical appearance, body satisfaction, state self-esteem, gender, TikTok

## Abstract

**Introduction:**

Fitspiration content promotes active, healthy lifestyles by advocating for healthy eating, regular exercise, and self-care. While Instagram’s impact on body image has been widely studied, limited research has explored TikTok fitspiration. This study examined how TikTok usage influences fit ideal internalisation, state self-esteem and perceptions of physical appearance, as well as gender differences in responses to fitspiration content on body satisfaction.

**Methods:**

A total of 274 participants (61.7% females; aged 18–62, *M* = 21.8, SD = 7.64) completed an online questionnaire assessing TikTok usage, fit internalisation and appearance-related perceptions. Participants then viewed three fitspiration videos, and their levels of body satisfaction and state self-esteem were measured before and after exposure.

**Results:**

Paired-samples *t*-tests revealed a significant decrease in body satisfaction post-exposure (*p* < 0.01, *Cohen’s d* = 0.47), while state self-esteem remained unchanged (*p* = 0.354, *Cohen’s d* = 0.023). A multivariate analysis of variance (MANOVA) showed that females’ state self-esteem and body satisfaction were significantly lower than males due to TikTok fitspiration content (*p* < 0.05). Multiple regression analyses revealed that among usage variables (e.g., frequency of use, posting habits, follower count), only the number of followees (β = 0.871, *p* = 0.020) and received “likes” (β = 1.449, *p* < 0.001) positively predicted fit internalisation, with no significant effect on appearance.

**Discussion:**

These findings highlighted the importance of educational interventions to counter the influence of TikTok fitspiration content. A potential implication of this study is the promotion of a shift from aesthetic ideals to physical capabilities.

## 1 Introduction

Body image refers to how satisfied an individual perceives and feels about their body’s shape, size, and appearance (Aimé et al., 2020; Grogan, 2006). Media, including social media, reinforce unrealistic, unattainable idealised beauty standards to their audience, emphasising appearance and attractiveness (Pryde and Prichard, 2022). These ideals typically reinforce thinness for women (Stewart and Ogden, 2020) and masculinity for men (Grogan, 2021). With over 5 billion active social media profiles worldwide (Kemp, 2024) and more than 56.2 million active social media users in the United Kingdom (Dixon, 2024a, 2024b), social media surpasses traditional media as a dominant source of body image content (Slater et al., 2017; Williams and Ricciardelli, 2014). Unlike traditional media, social media support users to generate and engage with highly curated, appearance-focused content (Burnette et al., 2017; Naslund et al., 2020).

Early body image research focused on platforms like Facebook, where interactions were more text-based (Verduyn et al., 2017). However, the emergence of Instagram—with its emphasis on visual content—shifted attention toward appearance-centric engagement (Limniou et al., 2021). This transition underscored how features such as ‘likes’, follower counts, and photo-sharing contribute to reinforcing idealised body standards and intensifying appearance-based social comparisons (Cohen et al., 2019; de Valle et al., 2021). Social comparison theory provides a robust framework for understanding the negative impact of social media on body image. Festinger (1954) initially introduced this theory, discussing how individuals are intrinsically motivated to evaluate themselves by comparing themselves to others, particularly in terms of appearance (Wheeler and Miyake, 1992). By applying this theory to social media, like Instagram, users are frequently exposed to idealised images of beauty posted by their peers or influencers. This can trigger unfavourable upward comparisons—where individuals perceive others as more attractive or socially approved (Hanna et al., 2017). These comparisons are often more potent than those triggered by traditional media for users, as they involve relatable figures from their peers rather than distant celebrities (Lee and Lee, 2021; Tiggemann and Zaccardo, 2018) and occur more frequently due to the accessibility and immediacy of social media platforms (Tiggemann and Zaccardo, 2015). Research has shown that such comparisons, especially when reinforced by curated content and quantifiable feedback, such as ‘likes,’ increased body dissatisfaction (Pedalino and Camerini, 2022; Rounsefell et al., 2020), well-being, and lower self-esteem (Sherlock and Wagstaff, 2019; Wang et al., 2017). A recent systematic literature review has identified social comparison as a strong mediator between social media use and body dissatisfaction, highlighting the cognitive and emotional processes that underlie social media’s effect on body image (Ryding and Kuss, 2020).

One strand of content particularly associated with these dynamics is fitspiration, a blend of *fitness* and *inspiration* that promotes appearance-focused fitness ideals (Tiggemann and Zaccardo, 2018). While it is considered a positive alternative to thinspiration, fitspiration often features highly aestheticised, toned physiques (Deighton-Smith and Bell, 2018) and can still provoke body dissatisfaction and appearance comparisons (Jerónimo and Carraça, 2022; Prichard et al., 2018). Fitspiration content is widely disseminated through Instagram (Cataldo et al., 2021), where users commonly share transformation narratives, fitness routines, dietary advice, and motivational imagery (Crossman, 2017). Although some posts offer health-positive messages (Robinson et al., 2017), the narrow aesthetic standards can undermine self-image (Alberga et al., 2018; Griffiths and Stefanovski, 2019). For example, content analyses have found that such imagery often conforms to narrow socio-cultural beauty standards—predominantly showcasing thin and toned physiques that may be unattainable for many viewers (Deighton-Smith and Bell, 2018; Goldstraw and Keegan, 2016).

This type of representation can undermine body image among users whose physical appearance diverges from these ideals, potentially fostering body dysmorphia (Prichard et al., 2018). Consequently, while fitspiration aims to promote healthy behaviours (Alberga et al., 2018; Raggatt et al., 2018), it may paradoxically contribute to body dissatisfaction (Carrotte et al., 2017; Holland and Tiggemann, 2017; Tiggemann and Zaccardo, 2015). High exposure to such content has been associated with decreased self-esteem (Alberga et al., 2018; Goldstraw and Keegan, 2016), heightened appearance-based comparison (Jerónimo and Carraça, 2022), and lowered mood (Limniou et al., 2021), particularly when influencers promote overly athletic and unrealistic body ideals. Although some studies suggest that fitspiration has no detrimental effects on body image or emotional state (Slater et al., 2017), the cumulative effect of frequent, subtle threats to body esteem may still erode self-perception over time (Griffiths and Stefanovski, 2019).

From a psychological perspective, these outcomes can be further explained through the self-objectification theory introduced by Fredrickson and Roberts (1997). This framework posits that individuals are socialised to adopt an external, observer’s view of their own bodies, which may begin to prioritise physical appearance over bodily function (Calogero, 2012; Daniels et al., 2020). Over time, such internalisation may lead to increased body surveillance, body shaming, and disordered behaviours, including negative mood states (Harper and Tiggemann, 2008), unhealthy weight management practices (Lepage et al., 2008), and a heightened drive for muscularity (Slater and Tiggemann, 2014).

While much of the existing fitspiration research focuses on young women (Holland and Tiggemann, 2017), emerging evidence suggests that men are increasingly engaging with this content and experiencing similar pressures to attain lean and muscular physiques (Perrin, 2015; Grogan, 2021). A third of fitspiration posts depict male bodies (Carrotte et al., 2017), reflecting its reach across genders. These patterns align with self-objectification theory, which posits that individuals internalise an observer’s perspective of their bodies, leading to chronic body surveillance, shame, and decreased self-worth (Calogero, 2012). As both men and women confront idealised body portrayals on platforms like Instagram, the psychological impacts—including reduced body satisfaction and heightened appearance monitoring—are increasingly recognised (Limniou et al., 2021; Jerónimo and Carraça, 2022). However, whether these effects generalise across platforms like TikTok or varying visual formats (i.e., videos) remains underexplored (Pan et al., 2023).

Since its launch in 2017, TikTok has amassed over 1 billion global users (Schellewald, 2023), offering an unrestricted platform for sharing short-form video content (between 15 and 180 s) (Omar and Dequan, 2020). Fitspiration content is widespread, with over 500,000 videos tagged under related hashtags by March 2024, and over 1 billion views by early 2022 (Pryde and Prichard, 2022). Despite this popularity, research on its psychological effects remains limited (Xu, 2024). Initial studies suggest that exposure to fitspiration TikTok posts heightens appearance-based comparisons, reduces body satisfaction, and lowers mood in both adolescents and adults (de Brabandere et al., 2025; Pryde and Prichard, 2022), mirroring patterns observed on Instagram (Prichard et al., 2020; Rounds and Stutts, 2021). However, few have examined these impacts across genders. The current study, therefore, investigates the influence of TikTok fitspiration on adult body image, focusing on gender difference, state self-esteem, body satisfaction, and internalisation of appearance ideals. Specifically, the hypotheses were to investigate whether:

*H1*: Exposure to TikTok fitspiration content will significantly affect participants’ state self-esteem and body satisfaction scores.

*H2*: Gender differences will significantly affect TikTok fitspiration exposure in state self-esteem and body satisfaction.

*H3*: TikTok usage (e.g., frequency, followers) will be positively associated with physical appearance comparison and internalisation of the fit ideal.

## 2 Methods

### 2.1 Study design

This study employed a within-subjects repeated-measures design examining the effects of TikTok fitspiration content. Independent variables included exposure type (neutral vs. fitspiration), TikTok usage, and participant gender. Dependent variables measured were state self-esteem, body satisfaction, physical appearance comparison, and fit internalisation. Participants viewed three neutral travel TikTok videos and three gender-matched fitspiration TikTok videos. Demonstrating different gender-matched stimuli to participants could help control for gender-related response biases, such as social desirability, which have been shown to influence ethical decision-making (Dalton and Ortegren, 2011). Neutral stimuli served as control conditions across all participants. The study was conducted in two phases: the first phase took place from October to December 2023, and the second phase took place from October to December 2024 (targeting male recruitment).

### 2.2 Participants

274 (females: 169 and males: 105) participants fully responded to an online questionnaire. All participants were at least 18 years of age, lived in the United Kingdom, and had a TikTok account. Participant ages ranged from 18 to 62 years (*M* = 21.8, *SD* = 7.64). All participants met the inclusion criteria: TikTok account holders aged 18 + living in the UK. Specifically, most of the participants were British (246 participants), while there were fewer EU citizens (16 participants) and non-EU international (12 participants) who resided in the UK.

*A priori* sample size calculation was conducted prior to data collection to determine the minimum number of participants required to estimate population-level proportions with acceptable precision. Specifically, the analysis focused on ensuring that key proportion estimates derived from questionnaire responses—such as the prevalence of particular attitudes or behaviours—would fall within a tolerable margin of error. Based on this calculation, a sample size of 274 yields a margin of error of ±5.88% at a 95% confidence level. This means that any proportion reported from the questionnaire (e.g., percentage of participants endorsing a given belief or behaviour) can be expected to vary by up to 5.88% in either direction, 95% of the time, if the study were replicated with similar samples. The margin of error was calculated to ensure adequate precision for descriptive estimates central to the study’s aims. While the term “power analysis” is often associated with hypothesis testing, in this context it was used to determine sample size for estimating proportions with a specified level of precision, rather than for detecting statistical effects.

After the ethical approval was granted by the University of Liverpool’s Research Ethics Committee, participants accessed the online questionnaire through social media and an internal recruitment scheme, using opportunity sampling as part of the recruitment process.

### 2.3 Questionnaire

The study utilised a 72-item online questionnaire, including TikTok videos, created and hosted on the web-secure survey Qualtrics platform. Participants engaged with both neutral (travel-themed) and fitspiration (fitness-focused) TikTok videos selected from publicly available content. The questionnaire design was informed by prior fitspiration research on Instagram (Limniou et al., 2021; Tiggemann and Zaccardo, 2015) and adapted for the TikTok platform. Gender-specific stimuli ensured consistent experimental exposure. All videos were sourced from public TikTok content. There were two sets of stimuli: 1. Neutral videos and 2. Fitspiration videos. The three neutral videos were posted on TikTok under ‘#travel’ and included relaxing videos; no people were contained within these travel videos. These were identical for male and female participants, with a total exposure duration of 47 s. The six fitspiration videos (three TikTok videos per gender) had been posted on TikTok under ‘#fitspiration’ or ‘#fitspo’, illustrating individuals in fitness clothing completing exercises in a gym environment while posing for the camera to present the fit parts of their bodies. Male participants viewed fitspiration content for a total of 54 s, while female participants were exposed for 48 s. A sample of screenshots of the stimuli is included in the Supplementary material.

Key psychological outcomes were measured using validated scales, including the Fit-Ideal Internalisation Scale, Physical Appearance Scale (PACS-R), and State Self-Esteem Scale (SSES). The Body Satisfaction Scale consisted of a single-item assessment. Reliability metrics for all adapted scales were high.

#### 2.3.1 Initial part of the questionnaire (demographic and TikTok usage items)

Initially, participants answered three demographic questions (i.e., sex, age, and ethnicity) and five TikTok usage items (i.e., average daily TikTok usage and the frequency with which they posted on TikTok). These questions were included in the initial part of the questionnaire to give insight into the participants’ TikTok usage habits and to gain a deeper understanding of the impact different usage factors have on individuals.

#### 2.3.2 Fit-Ideal Internalisation Scale (FIS)

The extent to which participants felt pressure from TikTok to comply with fit ideals was measured by the 11-item Fit-Ideal Internalisation Scale. Items within this scale were adapted by Sociocultural Attitudes Toward Appearance Scale-3 (SATAQ-3) (Thompson et al., 2004), stating “TikTok” instead of “TV or magazines” to enable its relevance to TikTok’s effects only. The scale required participants to respond to questions such as “I feel pressure from TikTok to exercise” and “I feel pressure from TikTok to diet.” Participants responded to each item on a 5-point Likert scale, assessing the degree of agreement toward the items (1- Definitely Disagree to 5- Definitely Agree). A maximum score of 55 could be achieved, with higher scores indicating increased pressure from TikTok to conform to fit ideals. Previous research has shown items in this scale had good reliability (*α* = 0.86; Thompson et al., 2004), and internal consistency for this scale in this study was excellent (*α* = 0.92).

#### 2.3.3 Physical Appearance Comparison Scale-Revised (PACS-R)

The 11-item Physical Appearance Comparison Scale-Revised, developed by Schaefer and Thompson (2014), was used to measure participants’ tendency to compare their physical appearance to the appearance of others. Participants were asked to indicate how often they have been engaged in physical appearance comparison by responding to questions including ‘When I’m out in public, I compare my physical appearance to the appearance of others’ and ‘When I’m at the gym‚ I compare my physical appearance to the appearance of others.’ using a 5-point Likert scale (1 = Never, 5 = Always). The score of this scale could range from 11 to 55, with higher scores indicating a greater tendency to engage in appearance comparison. The PACS-R has demonstrated excellent reliability in previous studies (*α* = 0.97; Schaefer and Thompson, 2014), and for this study, it has been the scale to have a high internal reliability (α = 0.95).

#### 2.3.4 State of Self-Esteem Scale (SSES)

Participants’ state self-esteem was measured twice, once after watching neutral travel TikTok videos and once after viewing experimental fitspiration TikTok videos. State self-esteem was measured using the 20-item State of Self-Esteem Scale (Heatherton and Polivy, 1991). This scale measured participants’ current self-esteem, meaning outcomes could fluctuate after exposure to different stimuli. Within the scale, participants had to answer questions such as ‘I feel good about myself.’ and ‘I feel self-conscious’. Participants’ level of agreement toward each statement was answered using a 5-point Likert scale (1 = Not at all, 5 = Extremely). Items 2, 4, 5, 7, 8, 10, 13, 15, 16, 17, 18, 19, and 20 were reverse-scored. A maximum score of 100 could be achieved, with higher scores indicating greater state self-esteem. Previous research established the scale to have excellent internal reliability (*α* = 0.92; Heatherton and Polivy, 1991). In the current study, the scale demonstrated excellent internal consistency following both neutral (*α* = 0.90) and experimental (*α* = 0.92) stimuli.

#### 2.3.5 Body Satisfaction Scale (BSS)

The extent to which participants were satisfied with their bodies was measured after viewing neutral travel TikTok videos and after experimental fitspiration TikTok videos through a single body satisfaction item. Participants were asked to rate their current satisfaction with their body appearance using a scale from 0 (not at all) to 100 (very great extent). Higher scores indicated greater satisfaction with their bodies at that moment.

#### 2.3.6 The last part of the questionnaire

Participants viewed experimental TikTok videos, which showed three fitspiration content videos depending on their input in the gender demographic question. After watching the fitspiration videos, participants responded to the same State of Self-Esteem Scale and the single-item body satisfaction question.

### 2.4 Procedure

Participants initially accessed the relevant participant information sheet, containing details such as the study’s aims, withdrawal process, anonymity, and data storage process, enabling them to make an informed decision about their participation and provide their consent. The questionnaire was completed in one sitting and included demographic questions, TikTok usage items, exposure to neutral and fitspiration videos, and repeated measures of psychological scales. All participants completed the survey in the same order. Upon completion, a debriefing form was provided with contact details for the research team and support resources, should any participant feel affected by the study materials. This procedure was approved by the University of Liverpool’s Research Ethics Committee.

### 2.5 Data collection and data analysis

Data were collected through an online questionnaire hosted on Qualtrics between October and December for two consequence years (2023 and 2024). Statistical analyses were conducted using SPSS version 28.0.1.1. Prior to analysis, data were screened for missing values, outliers, and violations of test assumptions.

To address Hypothesis 1, two paired samples t-tests were conducted to examine changes in participants’ state self-esteem and body satisfaction following exposure to fitspiration TikTok videos versus neutral travel content. The paired t-test statistical analysis was used because it can compare two related measurements from the same participants. Initially, it was explored whether there were any differences within the same participants.

For Hypothesis 2, a multivariate analysis of variance (MANOVA) assessed gender differences in state self-esteem and body satisfaction before and after fitspiration exposure. Gender was treated as a fixed independent variable, and post-hoc tests were performed where appropriate. By incorporating both pre- and post-exposure scores, the analysis captured not only baseline differences but also potential shifts attributable to the experimental manipulation. Where significant multivariate effects were observed, follow-up univariate ANOVAs and post-hoc comparisons were conducted to identify which specific dimensions—state self-esteem or body satisfaction—were differentially affected across gender groups. Effect sizes were reported to contextualise the magnitude of these differences.

For Hypothesis 3, two multiple regression analyses were conducted to investigate the predictive relationships between TikTok usage factors, physical appearance comparison, and fit ideal internalisation. Predictor variables included average daily TikTok use, posting frequency, and physical appearance comparison scores. These analyses have been conducted because physical appearance and fit-ideal internalisation were measured at a single time point and did not involve repeated measures, making regression an appropriate choice.

## 3 Results

### 3.1 Assessment of statistical assumptions

To ensure the validity of the repeated-measures ANOVA, several statistical assumptions were assessed.

Sphericity: Tested via Mauchly’s test, which indicated a violation (*W* = 0.75, *p* < 0.05). Greenhouse–Geisser corrections were applied to adjust degrees of freedom accordingly.

Normality: The normality of the residuals was evaluated using the Shapiro–Wilk test. The test results showed that the residuals were normally distributed (i.e., *W* = 0.99, *p* > 0.05). Additionally, Q-Q plots were inspected, and no significant deviations from normality were observed.

Homogeneity of variances: Levene’s test was conducted to assess the homogeneity of variances across groups. The test results indicated that the variances were equal (i.e., *F* = 1.23, *p* > 0.05).

Independence: Ensured through the study design, where each participant’s measurements were recorded independently.

Power analysis: According to G*Power (*α* = 0.05), with 274 participants, it has been detected small to medium effects (Cohen’s d = 0.3) with >95% power, which is more than adequate. Even for smaller effect sizes (e.g., Cohen’s d = 0.2), it still retained decent power (~80–85%).

### 3.2 Does the visual fitspiration exposure affect state self-esteem and body satisfaction?

A paired t-test was conducted to investigate the impact of fitspiration exposure on body satisfaction (Table 1). Revealing body satisfaction ratings were significantly lower before (*M* = 53.4, *SD* = ±20.74) and after viewing experimental fitspiration videos (*M* = 50.0, *SD* = ±21.94), *t* (273) = 7.344, *p* < 0.001, Cohen’s *d* = 0.44, 95% CI [0.32, 0.56].

**Table 1:** Paired t-test results indicating how exposure to fitspiration content affects state self-esteem and body satisfaction.

Dependent variable	Fitspiration Exposure	Mean	SD	t-test	*p*	Cohen’s d	95% Coefficient interval of the difference	
							Lower	Upper
State Self-Esteem	Before	53.4	20.74	*t* (273) = 7.344	<0.001	0.44	0.32	0.56
	After	50.0	21.94					
Body satisfaction	Before	61.9	12.89	*t* (273) = 0.374	0.354	0.023	−0.07	0.12
	After	61.7	14.1					

A second paired t-test was conducted to determine the effect of fitspiration exposure on state self-esteem (Table 1). Revealing there was no significant difference in state self-esteem scores before (*M* = 61.85, *SD* = ±12.89) and after viewing experimental fitspiration videos (*M* = 61.7, *SD* = ±14.1), *t* (273) = 0.374, *p* = 0.354, *Cohen’s d* = 0.023, 95% CI [−0.07, 0.12].

To mitigate potential Type I error, *p*-values were evaluated using the Benjamini-Hochberg procedure, with results remaining significant for body satisfaction.

### 3.3 Is there any difference between genders in state self-esteem and body satisfaction before and after the visual fitspiration exposure?

A multivariate analysis of variance (MANOVA) was conducted to compare body satisfaction and state self-esteem across gender and exposure conditions. Gender significantly affected the dependent variables, Pillai’s Trace = 0.059, *F* (4,268) = 4.231, *p* = 0.002, partial *η*^2^ = 0.059, 95% CI [0.020, 0.110]. Descriptive statistics per gender for each condition (before or after the fitspiration exposure) are provided in Table 2.

**Table 2:** Descriptive statistics for each dependent variable before and after exposure to fitspiration content, split by gender.

Dependent variable	Fitspiration exposure	Female		Male	
		Mean	SD	Mean	SD
Body satisfaction	Before	51.2	21.86	56.8	18.37
	After	47.2	22.96	54.3	19.48
State self-esteem	Before	59.6	13.23	65.5	11.45
	After	59.1	14.19	65.9	12.94

Because the MANOVA revealed a significant Gender × Condition interaction (before vs. after fitspiration exposure), follow-up univariate ANOVAs were conducted to identify which specific variables contributed to this interaction. These analyses revealed significantly lower scores for females compared to males across both time points. Table 3 presents the ANOVA results for gender and conditions. Specifically, there was a statistically significant difference between males’ and females’ body satisfaction before fitspiration exposure *F* (1,271) = 4.702, *p* = 0.031, partial *η*^2^ = 0.017, 95% CI [0.001, 0.055] and after fitspiration exposure F (1,271) = 6.804, *p* = 0.010, partial *η*^2^ = 0.024 95% CI [0.004, 0.067]. Also, there was a statistically significant effect of condition on state self-esteem before fitspiration exposure; females had significantly lower state self-esteem compared to males, *F* (1,271) = 14.608, *p* < 0.001, partial *η*^2^ = 0.051, 95% CI [0.020, 0.096]. Similar are the results for the state self-esteem after fitspiration exposure, with females having significantly lower state self-esteem than males, F (1,271) = 15.887, *p* < 0.001, partial η^2^ 0.055, 95% CI [0.022, 0.100].

**Table 3:** Gender (males and females) x condition interactions (before and after fitspiration exposure) and subsequent ANOVA significant results.

Gender (males vs females) x conditions	*F*	*p*	partial η^2^	95% Coefficient interval of the difference	
				Lower	Upper
Gender x Body satisfaction before Fitspiration Exposure	*F* (1,271) = 4.702	0.031	0.017	0.001	0.055
Gender x Body satisfaction after Fitspiration Exposure	*F* (1,271) = 6.804	0.010	0.024	0.004	0.067
Gender x State self-esteem before Fitspiration Exposure	*F* (1,271) = 14.608	<0.001	0.051	0.020	0.096
Gender x State self-esteem after Fitspiration Exposure	*F* (1,271) = 15.887	<0.001	0.055	0.022	0.100

To complement the primary analyses, a set of generalised linear models (GLMs) was conducted to further examine the interaction between gender and psychometric outcomes across conditions. These models, employing Gaussian link functions and robust standard errors, confirmed the significant effects observed in repeated measures. Specifically, gender remained a significant predictor of post-exposure body satisfaction (*β* = −3.02, *p* = 0.012, 95% CI [−5.38, −0.66]) and state self-esteem (*β* = −4.42, *p* < 0.001, 95% CI [−6.52, −2.32]), with females reporting lower scores than males. Model fit metrics (AIC/BIC) favoured inclusion of gender × condition interactions. Sensitivity analysis, excluding outliers > ± 2 SD, yielded consistent results across primary tests, indicating analytical robustness.

### 3.4 Does TikTok usage affect fit idealisation and physical appearance comparison?

Two multiple regressions were conducted to investigate the effect of TikTok usage on fit idealisation and physical appearance comparison. The first regression analysis explored the association between the TikTok usage factors with fit internalisation (*Mean* = 33.0, *SD* = ±10.17). Overall, the regression model was significant and predicted approximately 10.7% of the variance (Adjusted *R*^2^ = 0.090, *F* (5,268) = 6.422, *p* < 0.001). The importance placed on the number of likes received (*β* = 1.449, *p* < 0.001) and the number of people followed on TikTok (*β* = 0.871, *p* = 0.020) were significant positive predictors of fit internalisation. However, all the other TikTok usage factors were not significant predictors of fit internalisation: average TikTok screen time per day (*β* = 1.108, *p* = 0.066), frequency of posting on TikTok (*β* = −0.035, *p* = 0.965), and the number of TikTok followers (*β* = 0.116, *p* = 0.816).

A second multiple regression analysis explored the association of the TikTok usage factors with physical appearance comparison (*Mean* = 33.9, *SD* = ±10.72). The regression model was not significant (Adjusted *R*^2^ = 0.029, *F* (5,196) = 1.601, *p* = 0.160). None of the TikTok usage factors were significant predictors of physical appearance comparison: average daily TikTok screen time (*β* = 0.035, *p* = 0.957), posting frequency (*β* = 0.682, *p* = 0.432), the importance of likes received (*β* = 0.505, *p* = 0.240), the number of followers an individual had on TikTok (*β* = −0.012, *p* = 0.983), and the number of accounts followed on TikTok (*β* = 0.766, *p* = 0.062).

## 4 Discussion

The current study contributes to a growing body of literature exploring how TikTok fitspiration content influences individuals’ body satisfaction, state self-esteem, and internalisation of fitness ideals. While previous research has predominantly focused on social media platforms like Instagram affecting individuals’ views on body image and fitspiration, there are only a few studies which have investigated the potential effect of TikTok on fitspiration. TikTok’s rapid expansion and its algorithmically tailored content delivery present unique psychological dynamics warranting focused investigation (Zhou, 2024). The current study also explored gender differences in response to fitspiration content on body satisfaction and state self-esteem. Finally, this study additionally examined how TikTok usage patterns (e.g., number of followers, followees, and ‘likes’) relate to physical appearance comparisons and fit-ideal internalisation.

A key finding was that body satisfaction significantly decreased after exposure to TikTok fitspiration content. This finding aligns with existing literature linking TikTok fitspiration exposure with lower body satisfaction, especially among women (Pryde and Prichard, 2022). Drawing on social comparison theory (Festinger, 1954), this effect likely stems from upward comparisons with idealised, often unattainable physiques presented in such content (Möri et al., 2022; Krug et al., 2020; Aparicio-Martinez et al., 2019; Robinson et al., 2017). When exposed to hyper-curated portrayals of physical perfection, individuals may perceive themselves as lacking, leading to body dissatisfaction.

Gender differences were particularly evident. Women reported greater body dissatisfaction than men, a finding consistent with self-objectification theory (Fredrickson and Roberts, 1997), which posits that media representations emphasising appearance and sexuality—especially of women—encourage an externalised view of the body. Fitspiration content often objectifies female bodies (Bell et al., 2024), contributing to increased self-surveillance and lower body satisfaction among women (Carrotte et al., 2017; Deighton-Smith and Bell, 2018). Interestingly, males demonstrated a significant decrease in body satisfaction, diverging from traditional patterns and suggesting a shift in how male body image is shaped by social media. While women traditionally report higher body dissatisfaction due to societal beauty standards (Grogan, 2021), emerging literature suggests that men are increasingly exposed to muscular-ideal content, contributing to rising appearance concerns (Fatt et al., 2019; Donovan et al., 2020). Fitspiration frequently showcases lean and muscular male physiques, intensifying pressure to attain athletic ideals. This may lead to upward comparisons without the same degree of psychological resilience that many women have developed over time through repeated exposure and discourse around body positivity. Additionally, men may lack established coping mechanisms, rendering them more vulnerable to idealised portrayals and exacerbating body dissatisfaction. Beyond individual comparisons, sociocultural factors help explain this gender reversal. Contemporary masculinity increasingly incorporates aesthetic and performance elements, emphasising physicality as a form of capital and validation (Connor et al., 2021). TikTok’s algorithm-driven feed prioritises visually stimulating content, often reinforcing gendered ideals of physical perfection. Meanwhile, female body image concerns—while still prevalent—have benefited from broader cultural awareness and resistance movements, such as body neutrality and feminist discourse, potentially providing some protective effects (Griffin et al., 2022).

This study also revealed a significant gender difference in state self-esteem -“temporary fluctuations” in self-esteem (Heatherton and Polivy, 1991) - with women reporting lower levels than men after viewing both travel and fitspiration TikTok videos. Previous research found functionality appreciation to be positively associated with self-esteem (Alleva et al., 2017; Linardon et al., 2023). It is expected that individuals who focus more on their functionality and physical capabilities, rather than their physical appearance, positively reframe the way they feel about their bodies and themselves, having an unchangeable state of self-esteem (Alleva et al., 2015). Therefore, TikTok fitspiration videos present idealised and somehow unattainable body images, and viewers may not be inclined to appreciate their body’s functionality in response to this visual format, thus they do not protect their state self-esteem. However, research failed to exhibit clear gender differences between social media exposure and self-esteem (Saiphoo et al., 2020), opposing research determined that social media appears to have a stronger effect on female users’ self-esteem compared to males (Cingel et al., 2022). Cultural context further complicates this picture—Kapadia and Patki (2023) found increased self-esteem among Indian women exposed to fitspiration, highlighting the role of regional discourses. These mixed findings underscore the need for further research to confirm whether TikTok disproportionately affects women’s state self-esteem across cultures.

Finally, this study’s findings partially support the third hypothesis, showing that specific TikTok usage factors—such as the number of accounts followed and the importance placed on receiving ‘likes’—are positively associated with fit-ideal internalisation. No link was found between usage and physical appearance comparison. These findings align with media internalisation models, suggesting that individuals who actively invest in social media – through ‘likes’, follows, and content engagement – are more likely to adopt promoted fitness ideas (Seekis et al., 2020). Unlike prior studies that associated time spent on social media with appearance comparisons (Hanna et al., 2017; Holland and Tiggemann, 2016), this study highlights the importance of differentiating social media investment from mere screen time (Verduyn et al., 2017). Active psychological engagement—reflected in concern over likes and following habits—appears more predictive of internalisation outcomes than passive consumption. This study’s findings introduce the idea that the importance of having one’s posts reinforced through social media interaction, specifically through ‘likes’, leads to increased fit internalisation. Suggesting that when individuals ‘buy’ into the importance of their social media content being socially accepted by others, the pressure to comply with fit ideals increases, shedding light on the problematic nature of seeking ‘likes’ on posts. Additionally, the number of accounts followed emerged as a significant predictor of fit-ideal internalisation, likely due to increased exposure to fitness-related content (Donovan et al., 2020). The current study relied on self-report measures of usage to explain these TikTok usage discrepancies. Thus, future research should incorporate objective data (i.e., platform analytics) to enhance accuracy and assess how varying usage patterns influence body image.

As with all research, this study is subject to several limitations that should be considered when interpreting its findings. Firstly, the sample lacks global representation, comprising predominantly Western participants residing in the UK. This demographic skew limits generalisability across cultural contexts, as body image norms and social media consumption patterns may vary significantly. Future research should aim to recruit a more culturally diverse sample to assess the cross-cultural validity of TikTok fitspiration’s psychological impact. Secondly, while the age range included adults from 18 to 62 years, adolescents were excluded from the sample despite their high social media engagement. Given that approximately 91% of UK teens aged 15–16 are active social media users (Dixon, 2023), it is essential to explore how TikTok fitspiration may affect younger users, who may be particularly vulnerable to body image pressures during formative developmental stages.

Another methodological concern involves the artificiality of the exposure protocol. Although videos were sourced from live TikTok accounts, participants engaged with them in a controlled environment, with restricted time and without the typical interactive features such as liking, commenting, or scrolling. These interactive behaviours are central to the TikTok experience and contribute to emotional engagement and cognitive processing (Perloff, 2014). The brief exposure period—approximately 90 s—is also markedly shorter than average daily TikTok usage, which exceeds 52 min (Ceci, 2024), raising concerns about ecological validity. Future research should consider more naturalistic designs that better reflect habitual social media usage, including passive and active engagement patterns.

Additionally, all psychological and behavioural variables were measured using self-report instruments, which may be susceptible to social desirability bias, particularly around sensitive constructs such as body satisfaction and self-esteem. While self-report remains a common approach in psychological research, future studies should incorporate mixed-method or behavioural measures, such as physiological indicators, implicit attitudes, or platform usage analytics, to strengthen validity and reduce bias. The study also did not account for participants’ baseline body image concerns or pre-existing TikTok content preferences, which may have influenced their susceptibility to fitspiration content. Individual differences in body image, prior exposure to similar content, and algorithmic curation could have moderated the observed effects. Controlling for these variables, or assessing them before stimulus exposure, would yield more nuanced interpretations of fitspiration’s impact. Taken together, these limitations suggest caution in generalising the current findings. Further research is encouraged to adopt more inclusive sampling methods, longitudinal designs, interactive and ecologically valid stimuli, and objective usage metrics to deepen understanding of how TikTok fitspiration influences psychological well-being across diverse populations. Finally, future research would benefit from more targeted moderation designs and larger samples to robustly examine how gender may shape susceptibility to appearance-related social media influences, considering TikTok usage (e.g., frequency, follower count).

Despite these limitations, the present study clearly illustrated that brief TikTok fitspiration exposure leads to a decrease in body satisfaction, expanding previous findings to a new social media platform and an alternative content medium. Future research is needed to investigate the impact that longer-term TikTok fitspiration exposure has on body image. Furthermore, the results address the research gap surrounding gender differences in state self-esteem in response to fitspiration content, advancing the knowledge base concerning the influence of fitspiration exposure. Current findings also hold important practical implications, highlighting the need to educate TikTok users about the problematic effects that fitspiration exposure can have on their body image, and to, where possible, limit their exposure to this content. It is crucial to create intervention programmes that highlight the idealised nature of social media content, enabling individuals to critically evaluate the realism of the content they are viewing, potentially limiting its effects (Cho et al., 2022). Media outlets should take further action to promote healthy trends, demonstrating that a variety of body shapes and sizes can be fit, and to reduce content that consistently promotes unrealistic body standards. This could be achieved through influencers or sports personnel showcasing fit and healthy bodies that do not necessarily align with society’s narrow view on fitness. Moreover, more body-positive trends need to be promoted in the media, ensuring that individuality is praised and supported. Traditional media literacy programmes, which have previously shown some success in protecting individuals’ body image (Yager et al., 2013), could be expanded to encompass social media platforms, specifically fitspiration content. In addition, targeted interventions should help women safeguard their self-esteem by encouraging a focus on physical capabilities rather than appearance, fostering functionality appreciation as a protective factor against idealised media imagery.

## 5 Conclusion

To conclude, this study investigated the effects of fitspiration content on TikTok on body satisfaction, state self-esteem, physical appearance and fit internalisation while examining gender differences. To the best of the authors’ knowledge, no previous studies have explored these variables on TikTok under experimental conditions whilst also comparing genders. This study’s findings demonstrated that TikTok fitspiration exposure negatively impacts body satisfaction, extending previous findings to a new social media platform and a different visual format. Furthermore, the present study showed females’ state self-esteem to be lower than that of males both after neutral and fitspiration TikTok content, increasing the understanding of fitspiration exposure on gender. Finally, TikTok usage factors were positively associated with fit internalisation, highlighting how different social media interactions can influence appearance internalisation. With TikTok’s exponential growth over the last few years, it is essential to look further into these findings. With social media sites blocking the content of body shaming and influencers or sports personnel demonstrating that a variety of bodies can be fit and healthy. It is crucial to emphasise the significance of fitspiration on body satisfaction due to its multifaceted effects and the negative psychological and physical impacts of body dissatisfaction. Thus, further research is needed to see how long-term fitspiration exposure affects body image and whether a gender difference exists in response to this. Media literacy interventions must be expanded to incorporate social media, particularly fitness content, to try to create a less damaging social media experience for users.

## Data Availability

The datasets presented in this study can be found in online repositories. The names of the repository/repositories and accession number(s) can be found at: https://doi.org/10.5281/zenodo.14884388.
