# Supplementary material for: TikTok fitspiration and fitness ideal internalisation: gender differences in self-esteem and body satisfaction
Source: Front Psychol. 2025 Sep 17;16:1578510. doi: 10.3389/fpsyg.2025.1578510 (PMC12487955; doi:10.3389/fpsyg.2025.1578510)
Supplement: Supplementary file 1 [file Table_1.docx]

Supplementary Material

## Supplementary Figures


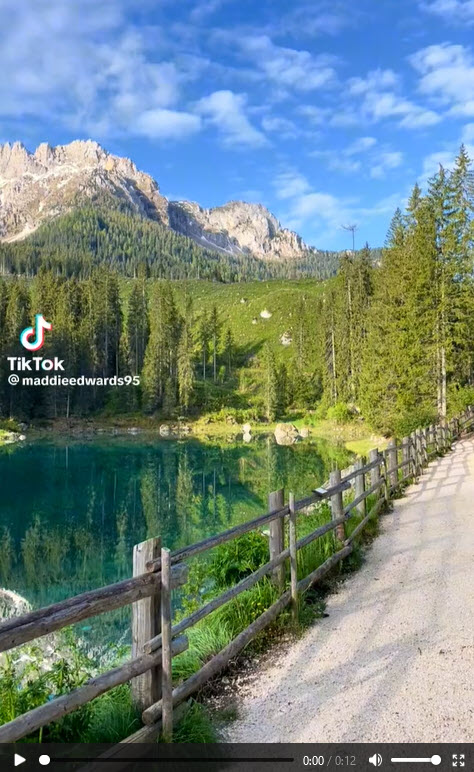


**Supplementary Figure 1.**
Screenshot from TikTok video by @maddieedwards95 (June 20, 2023), showing a relaxing countryside scenery.

**
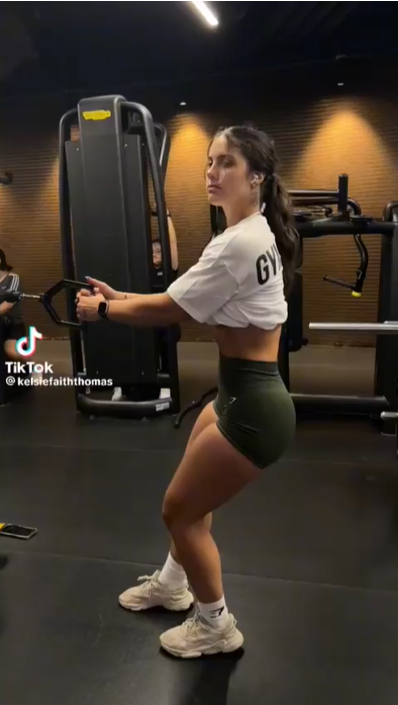
**

**Supplementary Figure 2.**

Screenshot from TikTok video by @kelsiefaiththomas (March 27, 2023), showing fitspiration activity for female participants.


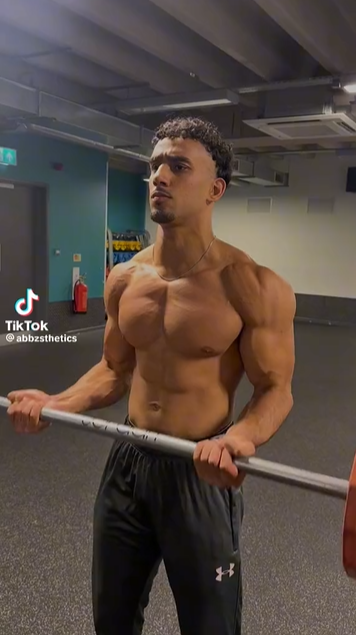


**Supplementary Figure 3.** Screenshot from TikTok video by @abbzsthetics (June 27, 2023), showing fitspiration activity for male participants.
